# *Rhodococcus erythropolis* as a host for expression, secretion and glycosylation of *Mycobacterium tuberculosis* proteins

**DOI:** 10.1186/s12934-017-0628-6

**Published:** 2017-01-19

**Authors:** Antonio J. Vallecillo, Cristina Parada, Pedro Morales, Clara Espitia

**Affiliations:** 1 Departamento de Inmunología, Instituto de Investigaciones Biomédicas, Universidad Nacional Autónoma de México, C.P. 04510 Mexico, D.F. Mexico; 2 Escuela de Medicina Veterinaria y Zootecnia, Facultad de Ciencias Agropecuarias, Universidad de Cuenca, C.P. 010220 Cuenca, Azu. Ecuador

**Keywords:** *Rhodococcus erythropoli*s, Recombinant glycoproteins, Posttranslational modification, *Mycobacterium tuberculosis*

## Abstract

**Background:**

Glycosylation is one of the most abundant posttranslational polypeptide chain modification in nature. Although carbohydrate modification of protein antigens from many microbial pathogens constitutes important components of B cell epitopes, the role in T cell immunity is not completely understood. There is growing evidence about the importance of these modifications in host bacteria interactions in tuberculosis. It is known, that the sugars present in some *Mycobacterium tuberculosis* glycoproteins play an important role in both humoral and cellular immune response against the pathogen. Since this modification is lost in the recombinant proteins expressed in *Escherichia coli*, it is fundamental to search for host bacteria with the capacity to modify the foreign proteins. Amongst the bacteria that are likely to have this possibility are some members of *Rhodococcus* genus which are Gram-positive bacteria, with high GC-content and genetically very close related to *M. tuberculosis*.

**Results:**

In this work, *apa*, *pstS1* and *lprG* genes that coding for *M. tuberculosis* glycoproteins were cloned and expressed in *Rhodococcus erythropolis*. All recombinant proteins were mannosylated as demonstrated by their interaction with mannose binding lectin Concanavalin A. In addition, as native proteins recombinants Apa and PstS1 were secreted to the culture medium in contrast with LprG that was retained in the cell wall.

**Conclusions:**

Together these results, point out *R. erythropoli*s, as a new host for expression of *M. tuberculosis* glycoproteins.

## Background

According to Worth Health Organization, tuberculosis claims about 2 million lives every year with almost one-third of the human population infected with *M. tuberculosis*, a microorganism that develops resistance to virtually all new drugs produced to fight it. Inadequate dosing and incomplete treatment regimens, together with the ability of the bacilli to cause latent infections that are tolerant of currently used drugs, have contributed to the rise of multidrug-resistant tuberculosis [1]. While intensive research efforts are addressing the pathogenesis, new drugs and vaccines are also necessary to explore into conceptual novel approaches for tuberculosis control. Studies of the role of *M. tuberculosis* molecules involved in host bacteria interactions identified a number of immunodominant mycobacterial proteins which undergo a process of posttranslational modifications such as glycosylation, lipoylation and methylation that provide important immunological properties [2–7]. Thus, these proteins are leading candidates for vaccine development. However, progress in this field has been hampered by the unavailability to obtain large quantities of recombinant modified extracellular proteins. At date, only a few *M. tuberculosis* proteins with posttranslational modifications have been expressed in *Mycobacterium smegmatis* and *Streptomyces lividans* since the recombinant proteins obtained in *E. coli* cannot be modified [2, 8–10].

Based on the high percentages of homology detected between putative lipoglycoproteins of *M. tuberculosis* and *Rhodococcus* spp. RHA1 as well as with a key enzyme for *O*-mannosylation, the *O*-mannosyl transferase, we envisioned *Rhodococcus* spp. as a candidate for expression of *M. tuberculosis* recombinant glycoproteins [7]. *Rhodococcus* spp. is a GC-high content bacterium, which metabolizes a wide range of compounds and represents a genus of considerable industrial interest [11]. In the present work, we investigated the feasibility of *R. erythropolis* as a host for the recombinant production of *M. tuberculosis* glycoproteins, Apa and the lipoglycoproteins PstS1 and LprG. All these proteins interact with molecules of innate immune system and play an important role in the induction of both cellular and antibody responses against *M. tuberculosis.* Apa is an Alanine/Proline rich antigen of 45/47 kDa and a potent inductor of T cell immune response which dependent of the *O*-mannosylation status [2, 3]. This protein together with PstS1 was the first glycoprotein described in *M. tuberculosis* [12]. PstS1 a 38 kDa is an immunodominant mannosylated lipoprotein (Lpp) and is also a phosphate transporter [13–15]. Disruption of *pstS1* gene reduces the in vivo multiplication of *M. tuberculosis* [16]. LprG a 27 kDa mannosylated Lpp, was found in the cell membrane of *M. tuberculosis* and *Mycobacterium bovis* [17, 18]. This protein is considered a virulence factor, since a knockout of *lprG* gene has proved to be attenuated in virulence in a mouse tuberculosis model [19]. In addition, all the proteins mentioned above, interact with the host through their glycan structures. Both Apa and LprG are ligands for macrophage and dendritic cell-specific intercellular adhesion molecule-3-grabbing non-integrin (DC-SIGN) [3, 20, 21] and Apa and PstS1 interact with macrophage mannose receptor (MR) [3, 22,]. Apa also binds to the pulmonary surfactant protein A, a member of C-type lectins from the innate immune system, this interaction is calcium- and mannose-dependent. Indeed all these molecules; DC_SIGN, MR and surfactant protein A have been shown to bind mycobacteria and facilitate their entry into phagocytes [20–23]. LprG and PstS1 interact with Toll-like receptors (TLR); however while the interaction of PstS1 through both TLR-2 and TLR-4 induces the activation of pathways, which play an essential role in tumor necrosis factor alpha (TNF-α) and interleukine 6 (IL-6) expression during mycobacterial infection [24], the interaction of LprG with TLR-2 inhibits Major histocompatibility complex class II antigen processing in human macrophages [25]. More recently, it was found that carbohydrate moieties were required for activation of TLR-2 by LprG to stimulate or inhibit T cell activation [3].

Because of the importance of *M. tuberculosis* antigens posttransduccionally modified, the availability of heterologous expression systems is a tool that will allow a deeper understanding of the role these proteins play in the pathogenesis of the disease.

## Methods

### Bacterial strains and culture conditions

*Escherichia coli* TOP10F′ (Invitrogen, USA) used for cloning was grown in Luria-Bertani broth (LB) (Difco, MI, USA). *R. erythropolis* strain L88, a lysozyme sensible mutant was grown to 26 °C in the same culture medium used for *E coli*. This bacterium was used as a host for recombinants thiostrepton induced (pTip-QC1) and constitutive (pNit-QC1) plasmids [26–28].

### Cloning of *M. tuberculosis* a*pa, pstS1*, and *lprG* codificant sequences in *R. erythropolis*

The complete coding regions of the *apa*, *pstS1* and *lprG* were amplified by PCR with the high fidelity DNA polymerase *Pfx* (Invitrogen) from *M. tuberculosis* H37Rv genomic DNA with the following oligonucleotide primers: ApaRhoFo: 5′-CATCAGGTGGACCCCAACTTGAC-3′ and ApaRhoRv, 5′-GA**GGATCC**GGCCGGTAAGGTCCGCTGC-3′ (*Bam*HI site in bold); PstS1RhoFo: 5′-AAAATTCGTTTGCATACGCTGTTGG-3′ and PstS1RhoRv5′-GA**GGATCC**GCTGGAAATCGTCGCGATCAAC-3′ (*Bam*HI site in bold); LprGRhoFo: 5′-CGGACCCCCAGACGCCAC-3′ and LprGRhoRv: 5′-GC**AAGATCT**GCTCACCGGGGGCTTCGTG-3′ (*Bgl*II site in bold). PCR products, *apa* (980 bp), *pstS1* (1127 bp) and *lprG* (714 bp) were cloned into the pTip-QC1 and pNit-QC1 vectors as follow: plasmids were digested with *Nco*I, 5′ *Nco*I overhangs were filled using T4 DNA polymerase (Thermo Scientific, USA) then, plasmids were digested with *Bgl*II and gel purified. Purified PCR product of *apa* and *pstS1* ware digested with *Bam*HI and cloned into the vectors to generate pTip-QC1-*apa* and pNit-QC1-*apa*; pTip-QC1-*pstS1* and pNit-QC1-*pstS1* respectively. PCR product of *lprG* was digested with *Bgl*II and ligated into the vectors to generate pTip-QC1-*lprG* and pNit-QC1-*lprG*. The identities and orientation of the inserts were confirmed by restriction analysis and DNA-sequencing. All the recombinant plasmids purified from *E. coli* were used to transform *R. erythropolis* L88 strain by electroporation as described elsewhere for mycobacteria [29]. Transformed cells were selected in LB agar plates supplemented with 34 μg/ml of chloramphenicol (Chl) and incubated at 26 °C.

### Expression and purification Apa, PstS1 and LprG C-terminal Hexahistidine-tagged proteins in *R. erythropolis*

A single *R. erythropolis* recombinant colony obtained of each pTip-QC1 and pNit-QC1 derivated vectors was grown overnight (ON) in 5 ml of LB broth supplemented with 34 μg/ml of Chl (LB-Chl) at 26 °C and 200 revolution per minute (rpm). The next day, small-scale expressions of recombinant Apa (rRhoApa), PstS1 (rRhoPstS1) and LprG (rRhoLprG) were performed by diluted ON cultures 1:100 in 50 ml of LB-chl. Cells transformed with constitutive pNit-QC1 recombinant vector were grown for 48 h at 26 °C at 200 rpm. Expression of recombinant proteins in cells transformed with pTip-QC1 vectors, were induced with 1 μg/ml of thiostrepton (Sigma) at 24 h of culture and then bacteria were grown for 24 more hours.

Cells were harvested by centrifugation at 3500×*g* for 15 min at 4 °C. Cell pellet was resuspended in lysis buffer (50 mM Tris–HCl, 50 mM NaCl, 1 mM DDT, pH 8.0), and bacteria was disrupted by sonication on ice during 15 min with cycles of 1 min and 1 min rest at 15–20% of potency in a Virsonic 550 sonicator (VirTis). After that, sample was centrifuged at 11000×*g* for 15 min at 4 °C to obtain the soluble extract (SE) and the insoluble fraction (IF). This fraction, was then washed twice with 1% Triton X-100 in phosphate buffered saline (PBS) and once only with PBS. After that, sample was solubilized in sample buffer (10 mM Tris–HCl, 50 mM NaCl, 5 mM imidazol, pH 8.0) with 8 M of urea and the mixture was stirred at room temperature (RT) for 20 min.

Cultures supernatant (CS) was precipitated with 90% of saturated ammonium sulphate solution at 4 °C, proteins were collected by centrifugation at 14000×*g* for 30 min at 4 °C. Obtained pellets were re-suspended in PBS and dialyzed against this same buffer for elimination of ammonium sulphate. Presence of recombinant proteins in the different obtained fractions was verified by sodium dodecyl sulfate–polyacrylamide gel electrophoresis (SDS-PAGE) and by Western blot assay with an anti-Hexahistidine Horseradish peroxidase antibody (anti-His_6_-HRP) (Roche).

rRhoApa, rRhoPstS1 and rRhoLprG were purified from clarified SE, IF and CS fractions using an AKTÄ Prime System (GE Healthcare) with 1 ml, Histrap^MT^ column (GE Healthcare). For SE and CS fraction after washing and equilibration of Ni–NTA column with sample buffer, sample was loaded and then the column was washed, with wash buffer (50 mM Tris–HCl, 50 mM NaCl, 25 mM imidazol, pH 8.0). Protein was eluted with elution buffer (10 mM Tris–HCl, 50 mM NaCl, 500 mM imidazol, pH 8.0) at 1 ml/min, by using a gradient (0–100% of elution buffer) and fractions displaying the recombinant proteins were pooled and dialyzed to eliminated the imidazol. In the case of IF, protein was purified in denaturing condition, all employed buffers contained 8 M urea and fractions displaying the recombinant proteins were pooled and dialyzed against decreasing urea concentrations. The presence of purified recombinant proteins was monitored by SDS-PAGE. The amount of protein was quantified by the modified Lowry method and proteins were stored at −70 °C until needed.

### Western blot assays and ligand blotting assay

Purified recombinant proteins from *R. erythropolis* SE, IF and CS fractions, were resolved on a 12% SDS-PAGE, transferred to a Polyvinylidene fluoride (PVDF) membrane and visualized by Coomassie brilliant blue R-250 staining. All membranes were blocked with 3% bovine serum albumin (BSA) in PBS at RT for 1 h. After three washes with PBS containing 0.05% Tween 20 (PBS-T), the presence of purified recombinant proteins from *R. erythropolis* cell fractions and CS were identified by Western blot assay with specific antibodies. Rabbit polyclonal antibodies raised against rRhoPstS1 and rRhoLprG [7] were diluted 1:500 in PBS-T and membranes were incubated for 1 h at RT in constant agitation. For specific detection of rRhoApa, membranes were incubated for 1 h with anti-Apa 6A3 monoclonal antibody (6A3 mAb) [30], diluted 1:500 in PBS-T/BSA. After several washes with PBS-T, membranes were incubated for 1 h with Protein A-HRP (Zymed) diluted 1:2000 in PBS-T for detection of polyclonal rabbit anti-sera and with anti-mouse IgG-HRP antibody (Invitrogen) diluted 1:2000 in PBS-T for detection of 6A3 mAb. Glycosylation of the recombinant proteins was determined by ligand blotting assay using the lectin Concanavalin A-HRP (Con A-HRP) (Sigma). Membranes were incubated with shaking ON at 23 °C with ConA-HPR diluted 1:1000 in incubation buffer (1 mM NaCL, 1 mM MgCL, and 1 mM MnCL) in presence or not of 0.3 M of methyl-α-d-mannopyranoside (Sigma). After incubation of membranes with the indicated primary and secondary antibody or ConA-HRP, membranes were washed with PBS-T, and developed with 3 mg/ml of 3,3-diaminobenzidine (DAB) (Sigma) in PBS with 1:1000 dilution of 30% of hydrogen peroxide.

## Results

### Expression and purification of rRhoApa

rRhoApa was similarly expressed from either *R. erythropolis* transformed with pTip-QC1-*apa* or pNit-QC1-*apa* vectors. Histidine-tagged proteins were found in SE, IF and CS fractions as detected with the anti-His_6_-HRP (Data not shown).

**Fig. 1:**
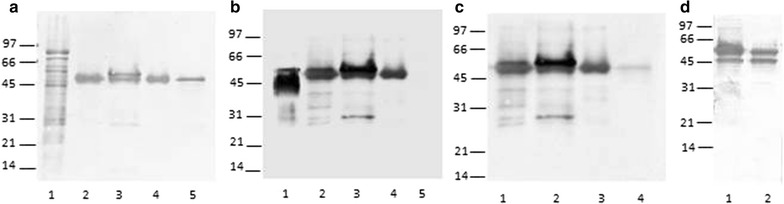
Analyses of purified rRhoApa from *R. erythropolis* transformed pNit-QC1-*apa* vector. **a** *M. tuberculosis* CS crude fraction and recombinant purified proteins blotted to PVDF membrane stained with Coomassie blue. *Lane 1*, *M. tuberculosis* H37Rv CS crude fraction. *Line 2*, purified protein from SE. *Line 3*, purified protein from IF. *Line 4*, purified protein from CS. *Line 5*, r*E*. *coli* Apa. **b**, **c** *Lines* are the same as **a**, but proteins were detected with Con A-HRP and with 6A3 mAb respectively. **d** *Line 1*, CS crude fraction from recombinant *R. erythropolis* and *line 2*, the flow through fraction from Ni-NTA column, both were detected with 6A3 mAb. Protein size markers are indicating on the* left*

Purification of protein was carried out by Ni–NTA affinity chromatography. Figure 1a, shows the Coomassie blue stained of purified protein from different fractions obtained from *R. erythropolis* transformed with expression vector pNit-QC1-*apa*, one band of 47 kDa was observed in rRhoApa purified from SE and CS (Fig. 1a, lines 2 and 4), a double band of 48/47 kDa was seen in purified protein from IF that could correspond to unprocessed and mature protein (Fig. 1a, line 3). All the purified proteins were recognized as a unique and well defined bands by Con A in ligand blotting assay (Fig. 1b) or in Western blot with the 6A3 mAb (Fig. 1c). However, when the CS crude fraction and the flow through fraction from the Ni–NTA column were incubated with 6A3 mAb, a double band of 45/47 kDa was detected (Fig. 1d, lines 1 and 2) in contrast with ConA or anti-His_6_-HRP which only detect the higher molecular mass band (Not shown).

### Expression and purification of rRhoPstS1

rRhoPstS1 was not expressed from *R. erythropolis* transformed with induced vector pTip-QC1-*pstS1*. Recombinant proteins obtained from bacteria transformed with constitutive pNit-QC1-*pstS1* vector were identified in SE, IF and CS fractions with anti-His_6_-HRP (Data not shown).

**Fig. 2:**
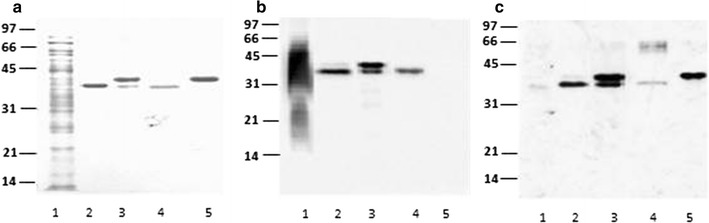
Analyses of purified rRhoPstS1 from *R. erythropolis* transformed with pNit-QC1-*psts1* vector. **a** *M. tuberculosis* CS crude fraction and recombinant purified proteins blotted to PVDF membrane stained with Coomassie blue. *Lane 1*, *M. tuberculosis* CS crude fraction. *Lane 2*, purified protein from SE. *Line 3*, purified protein from IF. *Line 4*, r*E*.*coli*PstS1. **b**, **c** *Lines* are the same as **a**, but proteins were detected with Con A-HRP and with rabbit polyclonal antibody raised against r*E.coli*PstS1. Protein size markers are indicating on the *left*

Figure 2a, shows the Coomassie blue stained of purified protein from the different fractions obtained from *R. erythropolis* transformed with expression vector pNit-QC1-*pstS1*. As native protein rRhoPstS1 was found in CS fractions. Two bands were observed in protein purified from IF, that could correspond to unprocessed and mature protein (Fig. 2a, line 4). Purified rRhoPstS1 from different fractions was recognized by Con A (Fig. 2b) and by rabbit polyclonal antibody raised against *E. coli* recombinant PstS1 (*rE.coli*PstS1) (Fig. 2c).

### Expression and purification of *M. tuberculosis* rRhoLprG

**Fig. 3:**
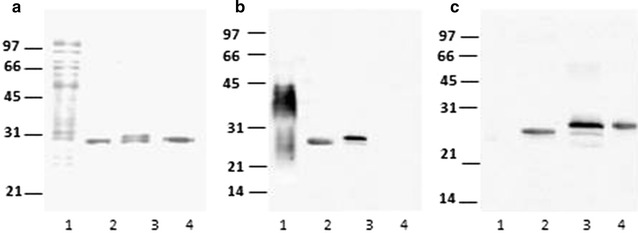
Analyses of purified rRhoLprG from *R. erythropolis* transformed pNit-QC1-*lprG* vector. **a** *M. tuberculosis* CS crude fraction and recombinant purified proteins blotted to PVDF membrane stained with Coomassie blue. *Lane 1*, *M. tuberculosis* CS crude fraction. *Line 2*, purified protein from SE. *Line 3*, purified protein from IF. *Line 4*, r*E*. *coli*LprG. **b**, **c**: *Lines* are the same as **a**, but proteins were detected with Con A-HRP (**a**) and with a rabbit polyclonal antibody raised against *rE.coli*LprG (**b**). Protein size markers are indicating on the *left*

rRhoLprG was similar expressed from either *R. erythropolis* transformed with pTip-QC1 or pNit-QC1 vectors (Results not shown). Proteins were detected with anti-His_6_-HRP in SE and IF but not in the CS (Not shown). In Fig. 3a, the Coomassie blue stained of purified proteins obtained from the different fractions is shown. Two bands were observed in protein purified from IF, that could correspond to unprocessed and mature protein (Fig. 3a, line 3). Both purified proteins from SE and IF were recognized by Con A (Fig. 3b) and by rabbit polyclonal antibody raised against *E. coli* recombinant LprG (*rE.coli*LprG) (Fig. 3c).

### Yields of purified proteins

Similar yields of purified recombinant proteins were obtained from SE and IF of rRhoApa and rRhoPstS1, in contrast with the lower amount obtained from CS of rRhoPstS1. Similar yields were also obtained with bacteria transformed with either pTip-QC1-*apa* or pNit-QC1-*apa* and the same was seen for pTip-QC1-*lprG* or pNit-QC1-*lprG* but with a very lower production of proteins from SE and IF proteins and not expression at all of rRhoLprG, protein from CS. rRhoPstS1 was not expressed from bacteria transformed with inducible pTip-QC1-*pstS1* vector. The yields of proteins are the average of 3 experiments (Table 1).

**Table 1:** Summary production of recombinant glycoproteins in *R. erythropolis*

Purified protein^a^ from *R. erythropolis* transformed with	Total purified protein (mg) from soluble extract	Total purified protein (mg) from insoluble fraction	Total purified protein (mg) from culture supernatant
pTip-QC1-*apa* ^b^	0.355 ± 0.07	0.334 ± 0.06	0.269 ± 0.042
pNit-QC1-*apa* ^c^	0.356 ± 0.09	0.349 ± 0.09	0.240 ± 0.06
pNit-QC1-*pstS1* ^d^	0.403 ± 0.04	0.399 ± 0.05	0.106 ± 0.012
pTip-QC1-*lprG* ^e^	0.011 ± 0.002	0.019 ± 0.01	NA
pNit-QC1-*lprG* ^f^	0.017 ± 0.004	0.016 ± 0.002	NA

*NA* no applicable

^a^ From 50 ml culture (^b^0.717 ± 0.155, ^c^0.663 ± 0.184, ^d^0.642 ± 0.07, ^e^0.585 ± 0.107, ^f^0.517 ± 0.102 g wet weight)

## Discussion

In this work, the coding regions corresponding to immature mycobacterial glycoproteins were amplified from *M. tuberculosis* H37Rv genomic DNA and cloned in both *R. erythropolis* pNit-QC1 (constitutive) and pTip-QC1 (thiostrepton inducible) expression vectors. The positive signal obtained with Con A in ligand blotting assays showed that Apa, PstS1and LprG the studied proteins were mannosylated, confirming this result that *R. erythropolis* possess the machinery to mannosylate proteins from *M. tuberculosis*. Although these proteins had been expressed in different host: all of them in *E. coli* [2, 7], PstS1 in *M. smegmatis* [8] and Apa in *M. smegmatis and M. bovis BCG* [2, 31], yields had been only reported for Apa produced in *S. lividans*. Vallin et al. [32] obtained 80 mg/l of purified rApa from CS and Gamboa-Suasnavart et al. [33] reported a higher production of CS rApa in coiled and in baffled flasks 7.44 ± 0.15 g/l than in conventional flasks 4.02 ± 0.08 g/l. From these results is clear that the production of rRhoApa from CS is lower that rApa obtained from CS of *S. lividans*. The evaluation of both recombinant proteins in a tuberculosis animal model will be important to define their antigenic and immunogenic potential.

Among the *M. tuberculosis* glycoproteins, Apa has been one of the most studied; this molecule is secreted to the culture medium as a double band of 47/45 kDa, through double Arginine translocase exportation system [34]. Their glycosylation sites determined by Dobos et al. [35] were located in Threonine (T), T_10_, T_18_ and T_27_ in N-terminus and in T_277_ in C-terminus. Furthermore, studies by Horn et al. [2] suggested that Apa double band is the result of proteolytic cleavage of the protein between Proline_275_ and T_276_, the calculated mass of the fragment devoid of mannose was 27,616 Da and could correspond to the C-terminal truncated peptide (1–275). Purified rRhoApa from CS was found as a unique band; however when the CS crude extract was incubated with 6A3 mAb, two bands of 47/45 kDa were seen. An explanation is that Apa could being processes in the C-terminus and due to the loss of the Hexahistidine-tag, only one band was purified. The not reactivity of the lower band with Con A in CS crude extract could be due to changes in glycosylation pattern as was observed from Apa recombinant expressed in *S. lividans* [7].

Both PstS1 and LprG are Lpp which are predicted to be localized on the cell wall, through their acyl chains. The N-terminal signal sequence of these molecules is distinguishable by the presence of a lipobox motif that includes an invariant Cysteine at the −1 position of the cleaved mature protein. However, as native, rRhoPstS1 was found in CS [7]. It is possible that as has been described for some Lpp of *Bacillus subtilis*, mycobacterial Lpp being consistently released from membrane through a proteolytic “shaving”. The most important determinant for Lpp release in *B. subtilis* seems to be on Glycine_2_ (G) position of the mature Lpp, whereas a Serine (S) on this position seems more important for membrane retention [36]. Interestingly, N-terminal sequence of native PstS1 released to CS showed a cleavage site between G_1_ and S_2_ position of the cleaved mature protein (Unpublished data).

It is worth of mention, that in addition to be very close genetically related to *M. tuberculosis* [11], *Rhodococcus* spp. in contrast with other actinomycetes have some advantages for expression of heterologous proteins such as: recombinant proteins are not affected by proteases activity and because the bacteria do not undergo a cellular differentiation, they grow very well in standard LB medium.

The importance of mannosylation in *M. tuberculosis* proteins is growing, and the role of glycans in host-pathogen interaction as well as the modulation of the immune response made them as important targets in the fight against tuberculosis.

## Conclusions

Results obtained concerning heterologous production of recombinant *M. tuberculosis* glycoproteins, demonstrated the potential of *R. erythropolis* as a valuable host for the production of recombinant proteins from *M. tuberculosis*.
