# Erratum to: *Rhodococcus erythropolis* as a host for expression, secretion and glycosylation of *Mycobacterium tuberculosis* proteins

**DOI:** 10.1186/s12934-017-0672-2

**Published:** 2017-04-19

**Authors:** Antonio J. Vallecillo, Cristina Parada, Pedro Morales, Clara Espitia

**Affiliations:** 1 Departamento de Inmunología, Instituto de Investigaciones Biomédicas, Universidad Nacional Autónoma de México, C.P. 04510 Mexico, D.F. Mexico; 2 Escuela de Medicina Veterinaria y Zootecnia, Facultad de Ciencias Agropecuarias, Universidad de Cuenca, C.P. 010220 Cuenca, Azu. Ecuador

## Erratum to: Microb Cell Fact (2017) 16:12 DOI 10.1186/s12934-017-0628-6

The authors of this published article [1] would like to add the following sentence to the ‘Acknowledgements’ section of the article [1]:

“We also thank the University program PASPA-UNAM for the scholarship granted to CE to carry out a sabbatical stay in Mucosal and Salivary Biology Division, King’s College, Guy’s Hospital, London, UK”.
